# Supplementary material for: MetaRibo-Seq measures translation in microbiomes
Source: Nat Commun. 2020 Jun 29;11:3268. doi: 10.1038/s41467-020-17081-z (PMC7324362; doi:10.1038/s41467-020-17081-z)
Supplement: Supplementary file 10 — Supplementary Data 7 [file 41467_2020_17081_MOESM10_ESM.zip › File2/Confidence_High_Taxonomy/231662_out.krona.html]

Javascript must be enabled to view this page.

members
magnitude
magnitudeUnassigned
count
unassigned
taxon
rank

231662\_out

3

2
3
superkingdom

1
phylum
1224

class
1
1236

order
1
135625

1
family
712

724
genus
1

1
species
729

SRS058730\_contig\_number\_20518

1
phylum
1239

1
class
186801

186802
1
order

1898207
species
1

SRS077849\_contig\_number\_contig-100\_20336.103138

phylum
1
32066

1
class
203490

203491
order
1

1129771
family
1

32067
1
genus


SRS076821\_contig\_number\_23587
1227267
species
1
